# Ethyl 3-benzyl­idenecarbazate

**DOI:** 10.1107/S1600536810048865

**Published:** 2010-11-27

**Authors:** Jin-He Jiang

**Affiliations:** a Microscale Science Institute, Department of Chemistry and Chemical Engineering, Weifang University, Weifang 261061, People’s Republic of China

## Abstract

In the title compound, C_10_H_12_N_2_O_2_, the dihedral angle between the mean planes of the aromatic ring and the side chain (r.m.s. deviation = 0.035 Å) is 18.23 (13)°. In the crystal, mol­ecules are linked by N—H⋯O hydrogen bonds, generating *C*(4) amide chains propagating in [010].

## Related literature

For related structures, see: Li & Jian (2010 ▶); Li & Meng (2010 ▶).
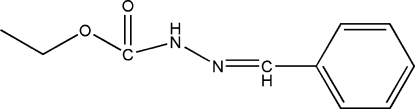


## Experimental

### 

#### Crystal data

C_10_H_12_N_2_O_2_
*M* *_r_* = 192.22
Orthorhombic, 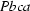

*a* = 11.309 (2) Å
*b* = 7.6693 (15) Å
*c* = 24.684 (5) Å
*V* = 2140.8 (7) Å^3^
*Z* = 8
Mo *K*α radiation
μ = 0.09 mm^−1^
*T* = 293 K
0.22 × 0.20 × 0.18 mm

#### Data collection

Bruker SMART CCD diffractometer
19439 measured reflections
2449 independent reflections
1147 reflections with *I* > 2σ(*I*)
*R*_int_ = 0.100

#### Refinement

*R*[*F* ^2^ > 2σ(*F* ^2^)] = 0.067
*wR*(*F* ^2^) = 0.206
*S* = 1.00
2449 reflections
127 parameters
H-atom parameters constrained
Δρ_max_ = 0.20 e Å^−3^
Δρ_min_ = −0.22 e Å^−3^

### 

Data collection: *SMART* (Bruker, 1997 ▶); cell refinement: *SAINT* (Bruker, 1997 ▶); data reduction: *SAINT*; program(s) used to solve structure: *SHELXS97* (Sheldrick, 2008 ▶); program(s) used to refine structure: *SHELXL97* (Sheldrick, 2008 ▶); molecular graphics: *SHELXTL* (Sheldrick, 2008 ▶); software used to prepare material for publication: *SHELXTL*.

## Supplementary Material

Crystal structure: contains datablocks global, I. DOI: 10.1107/S1600536810048865/hb5754sup1.cif

Structure factors: contains datablocks I. DOI: 10.1107/S1600536810048865/hb5754Isup2.hkl

Additional supplementary materials:  crystallographic information; 3D view; checkCIF report

## Figures and Tables

**Table 1:** Hydrogen-bond geometry (Å, °)

*D*—H⋯*A*	*D*—H	H⋯*A*	*D*⋯*A*	*D*—H⋯*A*
N1—H1*A*⋯O2^i^	0.86	2.04	2.885 (3)	168

Symmetry code: (i) 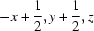.

**Fig. 1.**
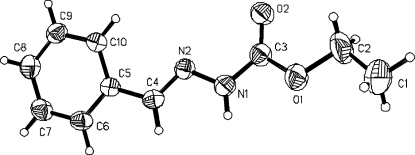
The structure of the title compound showing 30% probability displacement ellipsoids.

## supplementary crystallographic information

### Experimental

A mixture of benzaldehyde (0.01 mol) and ethyl carbazate (0.01 mol) was stirred in refluxing ethanol (20 mL) for 2 h to afford the title compound (0.095 mol, yield 95%). Colourless blocks were obtained by recrystallization from ethanol at room temperature.

### Refinement

H atoms were fixed geometrically and allowed to ride on their attached atoms, with C—H distances=0.97 Å, and with *U*_iso_=1.2–1.5*U*_eq_.

### Crystal data

**Table:**

C_10_H_12_N_2_O_2_	*D*_x_ = 1.193 Mg m^−^^3^
*M**_r_* = 192.22	Mo *K*α radiation, λ = 0.71073 Å
Orthorhombic, *P**b**c**a*	Cell parameters from 2449 reflections
*a* = 11.309 (2) Å	θ = 3.3–27.5°
*b* = 7.6693 (15) Å	µ = 0.09 mm^−^^1^
*c* = 24.684 (5) Å	*T* = 293 K
*V* = 2140.8 (7) Å^3^	Block, colorless
*Z* = 8	0.22 × 0.20 × 0.18 mm
*F*(000) = 816	

### Data collection

**Table:**

Bruker SMART CCD diffractometer	1147 reflections with *I* > 2σ(*I*)
Radiation source: fine-focus sealed tube	*R*_int_ = 0.100
graphite	θ_max_ = 27.5°, θ_min_ = 3.3°
φ and ω scans	*h* = −14→14
19439 measured reflections	*k* = −9→8
2449 independent reflections	*l* = −32→31

### Refinement

**Table:**

Refinement on *F*^2^	Primary atom site location: structure-invariant direct methods
Least-squares matrix: full	Secondary atom site location: difference Fourier map
*R*[*F*^2^ > 2σ(*F*^2^)] = 0.067	Hydrogen site location: inferred from neighbouring sites
*wR*(*F*^2^) = 0.206	H-atom parameters constrained
*S* = 1.00	*w* = 1/[σ^2^(*F*_o_^2^) + (0.1041*P*)^2^] where *P* = (*F*_o_^2^ + 2*F*_c_^2^)/3
2449 reflections	(Δ/σ)_max_ < 0.001
127 parameters	Δρ_max_ = 0.20 e Å^−^^3^
0 restraints	Δρ_min_ = −0.22 e Å^−^^3^

### Special details

**Table:**

Geometry. All e.s.d.'s (except the e.s.d. in the dihedral angle between two l.s. planes) are estimated using the full covariance matrix. The cell e.s.d.'s are taken into account individually in the estimation of e.s.d.'s in distances, angles and torsion angles; correlations between e.s.d.'s in cell parameters are only used when they are defined by crystal symmetry. An approximate (isotropic) treatment of cell e.s.d.'s is used for estimating e.s.d.'s involving l.s. planes.
Refinement. Refinement of *F*^2^ against ALL reflections. The weighted *R*-factor *wR* and goodness of fit *S* are based on *F*^2^, conventional *R*-factors *R* are based on *F*, with *F* set to zero for negative *F*^2^. The threshold expression of *F*^2^ > σ(*F*^2^) is used only for calculating *R*-factors(gt) *etc*. and is not relevant to the choice of reflections for refinement. *R*-factors based on *F*^2^ are statistically about twice as large as those based on *F*, and *R*- factors based on ALL data will be even larger.

### Fractional atomic coordinates and isotropic or equivalent isotropic displacement parameters (Å^2^)

**Table:**

	*x*	*y*	*z*	*U*_iso_*/*U*_eq_	
N2	0.26886 (18)	0.1974 (3)	0.64212 (7)	0.0581 (6)	
C5	0.3635 (2)	0.2155 (3)	0.72854 (9)	0.0531 (6)	
N1	0.2584 (2)	0.2687 (3)	0.59115 (7)	0.0683 (6)	
H1A	0.3045	0.3519	0.5811	0.082*	
C3	0.1754 (3)	0.2069 (4)	0.55722 (10)	0.0662 (7)	
O2	0.11238 (17)	0.0819 (2)	0.56498 (7)	0.0752 (6)	
C4	0.3463 (2)	0.2693 (3)	0.67226 (9)	0.0591 (7)	
H4A	0.3930	0.3582	0.6581	0.071*	
C6	0.4598 (2)	0.2798 (4)	0.75763 (10)	0.0663 (7)	
H6A	0.5139	0.3528	0.7406	0.080*	
O1	0.1713 (2)	0.3068 (3)	0.51302 (7)	0.0940 (7)	
C10	0.2827 (2)	0.1092 (3)	0.75513 (9)	0.0591 (7)	
H10A	0.2171	0.0668	0.7366	0.071*	
C8	0.3955 (3)	0.1299 (4)	0.83678 (10)	0.0730 (8)	
H8A	0.4062	0.1007	0.8730	0.088*	
C9	0.2993 (2)	0.0663 (3)	0.80883 (10)	0.0689 (8)	
H9A	0.2453	−0.0059	0.8263	0.083*	
C7	0.4757 (2)	0.2363 (4)	0.81131 (11)	0.0751 (8)	
H7A	0.5407	0.2790	0.8302	0.090*	
C2	0.0890 (4)	0.2517 (5)	0.47098 (14)	0.1325 (16)	
H2B	0.1114	0.1378	0.4573	0.159*	
H2C	0.0097	0.2435	0.4858	0.159*	
C1	0.0915 (5)	0.3756 (8)	0.42843 (15)	0.167 (2)	
H1B	0.0372	0.3408	0.4005	0.250*	
H1C	0.1699	0.3820	0.4137	0.250*	
H1D	0.0689	0.4878	0.4422	0.250*	

### Atomic displacement parameters (Å^2^)

**Table:**

	*U*^11^	*U*^22^	*U*^33^	*U*^12^	*U*^13^	*U*^23^
N2	0.0598 (13)	0.0575 (13)	0.0569 (11)	0.0006 (10)	−0.0028 (9)	0.0056 (9)
C5	0.0501 (14)	0.0482 (14)	0.0610 (13)	0.0036 (11)	−0.0004 (10)	0.0012 (10)
N1	0.0776 (15)	0.0659 (14)	0.0613 (12)	−0.0123 (11)	−0.0069 (11)	0.0136 (10)
C3	0.0709 (18)	0.0651 (18)	0.0625 (15)	0.0030 (15)	−0.0064 (13)	0.0077 (13)
O2	0.0767 (13)	0.0716 (13)	0.0773 (11)	−0.0072 (11)	−0.0111 (9)	0.0063 (9)
C4	0.0573 (15)	0.0547 (15)	0.0653 (14)	−0.0023 (12)	0.0026 (11)	0.0063 (11)
C6	0.0498 (15)	0.0712 (18)	0.0778 (17)	−0.0037 (13)	−0.0045 (12)	0.0056 (12)
O1	0.1229 (17)	0.0917 (15)	0.0675 (11)	−0.0224 (13)	−0.0252 (11)	0.0214 (10)
C10	0.0559 (15)	0.0573 (15)	0.0643 (15)	−0.0027 (12)	−0.0024 (11)	0.0006 (11)
C8	0.0762 (19)	0.077 (2)	0.0653 (15)	0.0128 (16)	−0.0065 (14)	0.0001 (12)
C9	0.0742 (19)	0.0658 (17)	0.0666 (15)	−0.0025 (14)	0.0069 (13)	0.0058 (12)
C7	0.0577 (17)	0.089 (2)	0.0783 (17)	−0.0010 (15)	−0.0175 (14)	−0.0027 (15)
C2	0.174 (4)	0.142 (3)	0.082 (2)	−0.046 (3)	−0.063 (2)	0.026 (2)
C1	0.172 (5)	0.231 (6)	0.096 (3)	−0.051 (4)	−0.045 (3)	0.054 (3)

### Geometric parameters (Å, °)

**Table:**

N2—C4	1.275 (3)	C10—C9	1.379 (3)
N2—N1	1.377 (2)	C10—H10A	0.9300
C5—C10	1.389 (3)	C8—C7	1.372 (4)
C5—C6	1.394 (3)	C8—C9	1.378 (4)
C5—C4	1.462 (3)	C8—H8A	0.9300
N1—C3	1.345 (3)	C9—H9A	0.9300
N1—H1A	0.8600	C7—H7A	0.9300
C3—O2	1.210 (3)	C2—C1	1.417 (5)
C3—O1	1.334 (3)	C2—H2B	0.9700
C4—H4A	0.9300	C2—H2C	0.9700
C6—C7	1.378 (3)	C1—H1B	0.9600
C6—H6A	0.9300	C1—H1C	0.9600
O1—C2	1.457 (4)	C1—H1D	0.9600
			
C4—N2—N1	114.8 (2)	C7—C8—H8A	119.9
C10—C5—C6	118.5 (2)	C9—C8—H8A	119.9
C10—C5—C4	121.8 (2)	C8—C9—C10	120.3 (3)
C6—C5—C4	119.5 (2)	C8—C9—H9A	119.9
C3—N1—N2	119.3 (2)	C10—C9—H9A	119.9
C3—N1—H1A	120.4	C8—C7—C6	119.8 (3)
N2—N1—H1A	120.4	C8—C7—H7A	120.1
O2—C3—O1	124.3 (2)	C6—C7—H7A	120.1
O2—C3—N1	126.3 (2)	C1—C2—O1	108.7 (3)
O1—C3—N1	109.3 (3)	C1—C2—H2B	109.9
N2—C4—C5	121.6 (2)	O1—C2—H2B	109.9
N2—C4—H4A	119.2	C1—C2—H2C	109.9
C5—C4—H4A	119.2	O1—C2—H2C	109.9
C7—C6—C5	120.8 (3)	H2B—C2—H2C	108.3
C7—C6—H6A	119.6	C2—C1—H1B	109.5
C5—C6—H6A	119.6	C2—C1—H1C	109.5
C3—O1—C2	116.0 (2)	H1B—C1—H1C	109.5
C9—C10—C5	120.3 (2)	C2—C1—H1D	109.5
C9—C10—H10A	119.8	H1B—C1—H1D	109.5
C5—C10—H10A	119.8	H1C—C1—H1D	109.5
C7—C8—C9	120.2 (3)		

### Hydrogen-bond geometry (Å, °)

**Table:**

*D*—H···*A*	*D*—H	H···*A*	*D*···*A*	*D*—H···*A*
N1—H1A···O2^i^	0.86	2.04	2.885 (3)	168

Symmetry codes: (i) −*x*+1/2, *y*+1/2, *z*.
